# Stakeholder Generated Ideas for Alternative School Food Provision Models in Australia Using the Nominal Group Technique

**DOI:** 10.3390/ijerph17217935

**Published:** 2020-10-29

**Authors:** Brittany J. Johnson, Dorota Zarnowiecki, Claire L. Hutchinson, Rebecca K. Golley

**Affiliations:** Caring Futures Institute, College of Nursing and Health Sciences, Flinders University, Bedford Park 5042, Australia; dorota.zarnowiecki@flinders.edu.au (D.Z.); claire.hutchinson@flinders.edu.au (C.L.H.); rebecca.golley@flinders.edu.au (R.K.G.)

**Keywords:** school food, nutrition, children, consensus process, nominal group technique, school lunches, lunch box, nutrition promotion, school meals, food service

## Abstract

Good nutrition is important for children’s learning, growth, and development, yet food intake during school hours does not align with recommendations. In Australia, most school children currently bring a packed lunch from home, but what if there was a different way? This project aimed to engage a diverse range of stakeholders to (1) generate, refine and prioritize ideas for novel models of food provision to Australian children within school hours, and (2) to determine and rank the potential barriers and facilitators to changing the school food provision system. This study used nominal group technique virtual workshops—three idea generation workshops (*n* = 21 participants) and one consensus workshop (*n* = 11 participants). School lunch prepared onsite was the top ranked food provision model option based on impact and achievability. Potential barriers (*n* = 26) and facilitators (*n* = 28) to changing the school food system were generated. The top ranked barrier and facilitator related to government support. This study highlights that there is an opportunity to explore partnerships and utilize existing skills and infrastructure to introduce a universal school-provided lunch model in Australia. The next steps should focus on building the business case capturing the social value of investing in school lunches, including considering parent-paid and subsidized options.

## 1. Introduction

Good nutrition during school years supports children’s health, growth, learning and development [1]. Primary school aged children attend school for up to six or more hours daily, and consume 23% to 44% of their daily energy intake in these hours [2,3]. Schools are a key health promotion setting to improve children’s health, including diet and activity patterns [4]. Despite this focus on schools as a setting to improve children’s nutrition, in Australia children’s intakes within school hours are not aligned with dietary guideline recommendations [5,6,7].

Most Australian children (~85 to 90%) bring a lunchbox packed at home [6,7], the contents of which have been similar for two decades. Analyses of primary-aged children’s school food intake from the National Nutrition Survey in 1995 identified low vegetable intake and an overrepresentation of energy-dense, nutrient-poor foods in the school setting [7]. While not a nationally representative sample, a study of children’s lunchbox contents in 2017 found that energy-dense, nutrient-poor foods contributed 39% of total energy packed, with 63% of children having two or more servings of these foods in their lunchbox, and only 20% of lunchboxes containing vegetables [5]. Evidence of differences in children’s lunch quality as determined by socio-economic position are mixed [5,6,7]. Food-insecure households are found to consume lower quality foods [8], with schools providing access to food relief school breakfast and lunch programs [9].

Australian families face the challenge of packing lunchboxes that are nutritious, safe and quick, and that their children will eat [10]. To date, nutrition promotion interventions have produced inconsistent improvements in lunchbox quality [11], most likely as the challenges of parental time needed to pack lunches and limited appropriate food items remain. If not bringing a packed lunch, children purchase food from a school canteen or tuck shop, which offer a range of ready-to-eat items and vary in management from paid canteen managers to parent volunteers or external companies [12]. School canteens face the challenge of making food available that aligns with nutrition guidelines, is financially viable and that children will eat [13]. School canteen guidelines have led to improvements in the nutritional quality of foods and drinks sold at school, but have not drastically changed children’s intake [14,15]. Over two thirds of primary school children’s canteen purchases are of less healthy choices, which are higher in saturated fat, total sugars and sodium [16]. Past interventions to improve school food have led to incremental changes, however reimagining Australian school food provision is an opportunity for transformative action to meaningfully improve children’s nutrition.

Food provision in schools differs internationally [12]. Lucas and colleagues [12] provide an overview of different approaches to school food provision policies in the UK, Sweden and Australia, highlighting that models range from universal free school-provided meals in Sweden to parent-provided lunches packed at home in Australia. The UK school food system varies, where children can bring a packed lunch or pay for a school meal, with free school meals available for young children living in low income households [12,17]. Many other countries have similar school food provision models with slight variations. For example, school food in the United States is commonly provided in a cafeteria, through the National School Lunch Program, with standards in place to guide nutritional quality [18]. A study was undertaken in Denmark to evaluate the impact of adopting school meals as a new food provision model in place of the current packed lunchbox model, finding that when consuming school meals children had higher intakes of several vegetables, legumes and fish [19]. Although various international models could be considered in Australia, research has not been undertaken to determine which model is best suited to the Australian context.

To generate ideas on how school food provision could be changed in Australia, it is essential to consult with key stakeholders, such as representatives from the education, health and social services, non-government, food industry sectors and parents. Engaging with stakeholders through the research process is increasingly recognized as an integral component in reducing research waste and increasing research with relevance to end users [20]. Co-design activities with stakeholders in the planning stages can include prioritizing research topics, setting research agendas, reviewing study plans and refining research designs and processes [20]. A recent example of co-design in the school setting is from Canada, where stakeholders were involved in developing a collaborative research agenda to address the school nutrition environment [21].

The current project aimed to engage a diverse range of stakeholders to (1) generate, refine and prioritize ideas for novel models of food provision to children within school hours, and (2) to determine and rank the potential barriers and facilitators to changing the school food system. The term ‘school food provision model’ refers to the way food in school hours is provided for children, for example via packed lunchboxes, school meals, etc.

## 2. Materials and Methods

### 2.1. Overview

This study used the nominal group technique method to engage key stakeholders to generate ideas and reach a consensus. The nominal group technique was first developed for program planning, and is an orderly, collaborative consensus process designed to generate, filter and prioritize ideas and solutions to questions in small groups [22]. The key benefits of this method are that the structured process minimizes group think, prevents the dominance of individuals, gathers a depth of ideas in a short period of time, and has a low participant burden and high rates of participant satisfaction [22,23,24]. The nominal group technique process involves a series of stages, including silent idea generation, round robin, clarification and collapsing, and voting, as described below [23]. This project included three idea generation workshops and one consensus workshop, conducted online in June and July 2020. Ethics approval was obtained from the Flinders University Social and Behavioural Research Ethics Committee (approval number 8468). Written notes were recorded directly by participants and the research team into online documents; workshops were not audio/video recorded.

### 2.2. Participants

Participant recruitment was undertaken via stakeholder groups’ email invitation, including a study flyer and link to the information sheet and consent form. A stakeholder mapping exercise was undertaken using a series of web searches to identify organizations and associations in the areas of education, health and social services, non-government organizations (e.g., food relief agencies, kitchen garden programs, school breakfast programs), food industry, design and creative industries, and parent advocacy. The study used a convenience sampling approach with snowball recruitment strategies, whereby the invited stakeholders were asked to pass on the invitation to others in their network. Written consent was obtained separately for the idea generation workshops and consensus workshops.

The participants completed a brief online survey and participated in a virtual 2 h workshop, on one occasion (idea generation or consensus only) or two occasions (both workshops). Due to the coronavirus pandemic 2019 (COVID-19) restrictions, the workshops were conducted virtually using Webex video conferencing (CISCO, Milpitas, CA, USA) and online Microsoft Word documents (Microsoft 365; Microsoft Corporation, Redmond, WA, USA). A maximum of seven participants has been recommended for the nominal group technique process, however previous studies have included between 2 and 14 participants [23]. Approximately 10 participants per workshop were scheduled to allow for a ~30% non-attendance. All workshops were facilitated by the same researchers, the workshop moderator (BJJ) and the workshop scribe and note recorder (DZ). Idea generation workshops were held in both school term time and school holidays to maximize involvement by participants in the education sector.

### 2.3. Nominal Group Technique Process: Idea Generation and Consensus Workshops

#### 2.3.1. Idea Generation Workshops

Prior to the workshop, the participants received via email the key questions: 1. “Imagine if school food was no longer lunchboxes and canteens, what would this look like?”; and 2. “What potential barriers and facilitators can you imagine to changing the school food system?”. Question one was phrased to encourage participants to think of the ideal situation and promote creativity [25].

**Figure 1:**
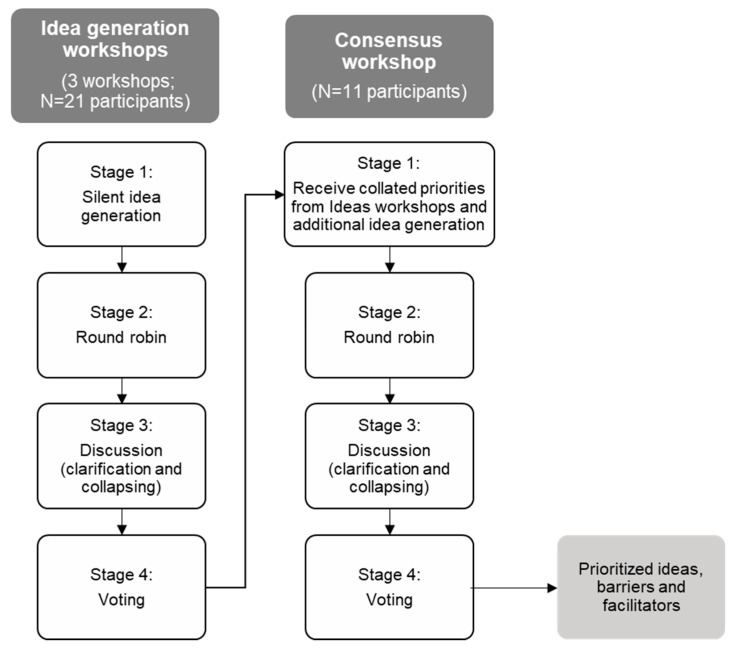
Flowchart of nominal group technique procedure for idea generation and consensus workshops.

During the workshops, the background of the current Australian school food context and ideas process was presented, before working through the four-stage nominal group technique process (see Figure 1) for question one, which was repeated for question two. The term ‘idea’ is used to refer to ideas for food provision models, and potential barriers or facilitators. The four-stage nominal group technique process followed that of previous studies, with adjustments for the online workshop format [25,26].

Stage one: Participants silently brainstormed and recorded their ideas for the question posed on paper or a blank word document.

Stage two: Participants shared one idea at a time, round-robin style, as guided by the researcher moderating the workshop. Ideas were recorded by a researcher scribing in a shared online word document visible to participants. If participants had no new ideas to add they were able to ‘pass’; multiple participants stating pass prompted when to conclude the round. Participants then typed a fuller description of the ideas they provided into a shared online word document. For question two, the idea sharing process involved all participants typing their ideas for potential barriers and facilitators directly into a shared online word document forming a collective list.

Stage three: The group discussion process allowed participants to ask clarifying questions and to build on other participants’ ideas. The moderator summarized each idea and moderated the discussion, repeating this process for all ideas. Participants were able to contribute to the discussion verbally or by using the videoconferencing chat function. This stage did not involve evaluating ideas, but rather ensuring participants understood each of the ideas. Participants were also asked whether there were duplicate ideas that could be collapsed prior to voting.

Stage four: Participants voted for their top three ideas based on importance, allocating 3 points to their most preferred idea, 2 points to their next preference and 1 point for their third preference. Voting used a shared online word document with participants typing their votes directly into the public voting ballot.

At the end of the ideas process, the total votes were tallied by the researchers and the top three ideas for each question were relayed to participants. Information was also provided about the next steps, including an invitation to take part in the consensus workshop.

Three participants who were unable to attend the scheduled workshops completed a written submission using the same data collection forms as used in the workshops but did not take part in the discussion or voting process. All ideas for food provision models, potential barriers and facilitators were collated from the three ideas workshops and the written submissions to take forward to the consensus workshop.

#### 2.3.2. Consensus Workshop

The consensus workshop aimed to bring together ideas from the separate idea generation workshops and provide an overall list of prioritized ideas. The consensus workshop followed a similar process to the idea generation workshops and used the same two questions. Ideas for food provision models from the idea generation workshops were collated and grouped into common themes by the research team, and circulated to consensus participants prior to the workshop and presented in Stage one of the ideas process. Participants were asked if there were further groupings of ideas or if there were themed ideas that should be separated. At this stage participants could also add any new ideas. The workshop then followed the same three stages of round-robin, discussion and voting as the idea generation workshops. Voting used an online survey tool (Qualtrics^®^, Provo, UT, USA), rather than a shared online word document, and private voting for food provision ideas was undertaken twice. Participants voted for ideas based on impact and were asked to consider the likely benefits to children, families and schools, reach to all children, and ideas less likely to have negative or unintended consequences. Participants also voted for ideas based on achievability by ease to implement, more support from key stakeholders, and lower costs. The same 3-point, 2-point, 1-point system was used when voting for the impact of ideas, the achievability of ideas, the potential barriers, and the potential facilitators. At the end of the ideas process, the total votes were tallied and the top three ideas for each question were relayed to the participants.

### 2.4. Analysis

The votes were recorded and total scores were tallied during the workshop (DZ). Following the workshop, the scores were checked for accuracy by a second researcher (BJJ) and the top three ranked priorities were circulated to workshop participants via email. After the workshop, voting frequency, that is, the number of participants who voted for an idea, and the relative importance score were also calculated (BJJ). Relative importance scores were calculated by the tallied score for each idea divided by the maximum points for the group, multiplied by 100.

## 3. Results

### 3.1. Participants

In total, 21 people participated in the idea generation workshops, attending one of three workshops (workshop one: *n* = 8; workshop two: *n* = 5; workshop 3: *n* = 5) or via written submission (*n* = 3). Participants were primarily from the education (*n* = 11) and/or non-government (*n* = 9) sector. Experience in the sector ranged from less than 1 year to 47 years, with a median of 11 years of experience.

In total, 11 people participated in the consensus workshop, 9 of whom had participated in the idea generation workshops. The participants were from a range of sectors, with experience in the sector ranging from less than 1 year to 40 years, with a median of 3.5 years of experience.

Table 1 presents a summary of participant characteristics from the idea generation workshops and the consensus workshop. Across both the idea generation workshops and the consensus workshop, participants brought a breadth of relevant experience, including working with socio-economically disadvantaged populations and Aboriginal communities, food technology, teaching, food and nutrition programs, food relief and canteen management. Many of the participants were also parents themselves (~65%).

### 3.2. Idea Generation Workshops Summary of Results

Participants in the idea generation workshops identified a total of 34 ideas for alternative food provision models for the Australian context. Many of the ideas centered on similar concepts and themes, and were synthesized to 11 discrete food provision models. The ideas included food cooked onsite, student involvement in preparing meals, healthy snack vending machines, bulk-prepared meals delivered to schools, and individual food boxes (Supplementary Table S1).

The participants generated a total of 53 potential barriers and 49 potential facilitators to changing the school food system. After collapsing duplicate ideas, there were 24 discrete potential barriers and 30 potential facilitators to take into the consensus workshop.

### 3.3. Consensus Workshop Results

#### 3.3.1. Food Provision Model Ideas

Consensus workshop participants collapsed two food provision model ideas—school lunch prepared onsite and student-led group food preparation. The participants also removed one idea—celebrity recipe branding—that was considered a strategy rather than a standalone food provision model. As no additional ideas were added, this resulted in nine food provision models taken forward to voting.

Table 2 presents the prioritized and ranked food provision models from the consensus workshop. ‘School lunch prepared onsite’ was ranked the top food provision model in terms of both likely impact (relative importance score 44%) and likely achievability (relative importance score 26%). This model was described as food prepared onsite by a cook/team of kitchen staff for a sit-down meal, based on a rotational menu reflecting seasonal produce, minimally processed foods and dishes representing different cultures. The additional components of this model included using canteen facilities, subsidized fees dependent on family income, teachers eating the same foods whether siting with students or in a different location, and optional student involvement in food preparation.

The second ranked food provision model based on likely impact was a ‘community restaurant’ (relative importance score 18%), whereas when considering achievability, ‘school lunch prepared off-site (centralized)’ (relative importance score 20%) was ranked second. The ‘community restaurant’ model was characterized as purpose-built restaurants to service multiple schools, seniors’ centers and community groups, where there could be interaction between students and other community members. When discussing the community restaurant idea participants noted the broader potential benefits of such a model for the community as well as students, for example, learning road safety when walking to the venue. ‘School lunch prepared off-site’ involved centralized food production by dedicated food preparation staff, and delivery in bulk to be served to students on school grounds.

‘Student/self-food preparation’ was held equal third rank and similar relative importance scores for impact (15%) and achievability (18%). This model would involve students selecting and preparing their own lunch (e.g., salad bowl/sandwich) and snacks before school or in the classroom, at a food creation station or ‘mini supermarket’. The choices would be guided by dietary guideline recommendations and the items may be semi-prepared but minimally processed (e.g., carrot sticks, cubed cheese). When discussing this idea, the participants noted this model could include food for before and after school for students from disadvantaged backgrounds.

#### 3.3.2. Potential Barriers and Facilitators to Changing the School Food System

Two additional barriers to changing the school food system were added by participants in the consensus workshop, with a total of 26 potential barriers taken forward to voting. See Supplementary Table S2 for the full list of barriers. The top five ranked barriers are presented in Table 3. The barriers receiving the majority of the votes and therefore the highest relative importance scores were ‘political barriers and lack of government support’ (27%), ‘financial barriers and cost involved (e.g., staffing, products/service)’ (24%) and ‘change in infrastructure and equipment required (e.g., kitchen, dining)’ (20%).

During the consensus workshop, three potential facilitators to changing the school food system were condensed, and one additional facilitator added, resulting in a total of 28 potential facilitators taken forward to voting. See Supplementary Table S3 for the full list of facilitators. Additional details were added to three facilitators to reflect key aspects highlighted by participants in the group discussion stage. The top five ranked facilitators are presented in Table 4. The top facilitator receiving the most votes and therefore the highest relative importance score was ‘government support including cross agency and all political parties being committed’ (41%). This was followed by ‘tailored approach with variations for every type of school (e.g., small schools, regional schools with no kitchen)’ (14%) and ‘linking with external organizations, associations, sponsors or philanthropists working in the school food or nutrition space’ (14%), with an equal ranking.

## 4. Discussion

This project brought together a range of diverse stakeholders to generate, refine and prioritize food provision model ideas for re-imagining school food provision in Australia. Ideas for school food provision models, together with identifying barriers and facilitators, were co-produced with representatives from the education, health and social services, non-government and food industry sectors, and parents. School lunch prepared onsite was the top ranked food provision model when considering the likely impact and achievability of the model. The most important ranked barrier and facilitator was government support. Other prioritized barriers to address when changing the school food system included the financial barriers and the infrastructure and equipment needed. However, the prioritized facilitators highlight opportunities to mitigate barriers by exploring linking with existing organizations working in the school food or nutrition area, and having tailored approaches to school food provision models.

School lunch prepared onsite was the clear top ranked food provision model. This model was ranked by stakeholders as the model likely to have the largest impact in terms of benefits to children, families and schools, have the greatest reach, and be the least likely to have unintended consequences. In addition, this model was prioritized as the most achievable, when considering how easy it would be to implement, as it was deemed likely to garner significant support from key stakeholders and have lower associated costs. The study findings highlight that there is initial support for a universal school-provided model for Australian schools, where universal refers to all children consuming school-provided lunches, rather than an optional addition to lunches packed from home. A universal approach provides a sustainable and equitable approach to support food-insecure families, and an alternative to the current reliance on the food relief or food rescue sector [27]. This model could expand and innovate from the canteen system—a system that currently services around 15% of students a day through student- or parent-purchased meals or snacks [7]. A review exploring the adoption and implementation of school canteen policies in Australia found that despite having nutrition policies, school canteen staff still face several challenges in meeting nutrition recommendations, including impacts on profits and lack of support from parents and students for healthy choices [13]. A universal approach to school lunches can help overcome many of the challenges canteen staff face by embedding food preparation staff as an integral part of the school community and improving financial viability through increased demand. As less than 1% of children are meeting dietary guideline recommendations [28], there is likely to be universal benefit to Australian school children. However, we predict that these benefits will be greatest in families experiencing disadvantages, who may not have access to sufficient food during school hours or who rely on school food relief programs. When exploring the feasibility of a universal school lunch model in Australia, it is essential to consider how this could lessen the socio-economic divide and capitalize on canteen staff expertise.

While government-funded schools emphasize the importance of gaining the education department’s backing, government support to change the school food system is much broader. Food is an essential human right, with appropriate nutrition providing children with the chance to grow, learn and develop to their potential [29], hence school food is important for children’s health and is of interest to the health department. In addition, school food is of relevance to the primary production industry and welfare sectors. Participants in the workshops discussed the importance of cross agency work, across all of the government areas, and all political parties being committed to the long-term benefits of all Australians. A suggested facilitator included having a position within government to lead the school food agenda. There are opportunities to advocate for changing the system, including communicating the link between nutritious food and learning, social skills, and health and wellbeing outcomes, and how the different sectors can work together and mutually benefit. The stakeholders participating in this study demonstrated initial interest in transforming the way food is provided in Australian schools, and albeit influenced by sampling bias, these stakeholders can act as champions for change. We can work with existing groups, such as the Federation of Canteens in Schools (FOCIS; www.focis.com.au), to help advocate for change and build government awareness. There remains a need to continue to build the evidence and quantify the potential benefits, including the social value of implementing an alternative school food provision model to provide government decision makers with the necessary information to back the concept.

The stakeholder-generated barriers and facilitators to changing the Australian school food system provide substantial insights and direction to continue to build the business case for such a change. Importantly, there are several prioritized facilitators that can be explored to address key barriers. For example, school canteen kitchens could be repurposed to prepare school lunches onsite, and we can draw on international examples such as in Japan where classroom tables are reorganized for shared mealtimes [30], to overcome some of the infrastructure challenges. In addition, there are opportunities to partner with retailers, primary production, suppliers and food rescue agencies to minimize ingredient costs to schools. At the same time, school-provided lunches open a new market to guide demand for foods from the five food groups and industry innovation. Parent-paid options could be explored as an approach to minimizing government or school expense, as a subsidized user-paid system where the cost tradeoffs may be of interest to families [31]. We have begun to highlight a range of existing opportunities that could be leveraged to mitigate several potential barriers to changing the school food system, all of which can be explored further with the relevant stakeholders.

This study provides the first step towards reimaging school food in Australia. More work can be undertaken to extend the co-produced findings from the current study to continue to build the business case to advocate for change. As previously mentioned, further research is needed to determine the social value of implementing a school lunch system in Australia; this work should extend beyond the immediate nutritional, educational and health benefits of children to consider the broader benefits to families, schools, the community and society. The extensive list of potential barriers and facilitators to changing the school food system provide guidance for future research and practice to consider how we can minimize and overcome such barriers and what strengths we can draw on along the way. It is important that future work in this space engage with funders, families and the school community to continue to build upon the enthusiasm and momentum of participants in the current study. Of note, future research is needed with school students to determine whether the proposed alternative school food provision model is acceptable, and to refine how this model could operate.

Several key strengths centered on the study design. The nominal group technique approach provided a richness in the data through insights obtained from the group discussion that would not have been possible with alternative methods, such as a Delphi survey. We were able to engage with representatives from a range of sectors, including from education, non-government, food industry and services and health promotion sectors, and parents. The online workshop delivery also enabled stakeholders outside South Australia to participate in the study. Using co-design methods ensured this research was centered on stakeholder needs and perspectives. An unanticipated benefit of this study design was the opportunity to connect with potential partners. Participating in the interactive workshops provided a two-way learning experience.

The study limitations resulted from the convenience sampling and snowball recruitment, whereby we did not have representation from all states and territories, including canteen representation from New South Wales and Queensland, which have the largest number of school canteens. Stakeholders volunteering for the study likely represented those with a vested interest in nutrition and school food; yet this did highlight that there are several champions in various sectors. Given the high level of intellectual input needed by stakeholders at this stage of the research, school students were not recruited for this study. Students will need to be consulted to determine the acceptability of the prioritized alternative school food provision models in future research. The consensus workshop did not include education and parent representatives; this may have been due to the consensus workshop timing, with the session being held in school term. However, these groups were represented in the idea generation workshops, and the top-ranked priorities were comparable between the two phases. Many of the participants were also parents themselves, which may have been a conflict of interest. Finally, the online workshop delivery, due to COVID-19 related restrictions, at times meant that researchers were not able to see all non-verbal cues, and needing to ‘unmute’ may have limited participation. Conversely, the online mode removed geographical limitations and did allow for written chat, as well as verbal discussion that may have led to increased contributions by participants who might have been less vocal in an in-person workshop.

## 5. Conclusions

A diverse group of stakeholders in the Australian school food system generated ideas for how school food provision could be done differently, providing initial support for a universal school-provided lunch model in Australia. Several prioritized facilitators can be explored to address the key barriers identified to changing the school food system. There are opportunities to explore the repurposing of existing canteen infrastructure and capitalizing on the expertise of food preparation staff. Ultimately, to change the school food system, widespread government support is needed. A key step towards gaining this support is to first build the business case, capturing the social value of investing in school lunches. Both parent-paid and subsidized options to funding such a model should be explored. Moving forward, partnerships between government, non-government organizations, food supply and researchers offer an avenue to transform the school food system and ensure all Australian children eat well at school.

## Figures and Tables

**Table 1:** Background characteristics of workshop participants.

Characteristic	Idea Generation Workshops (*n* = 21 ^1^)	Consensus Workshop (*n* = 11)
**Sector ^2^**		
Education	11	5
Health services	4	3
Social services	0	1
Non-government/not for profit	9	7
Food industry, service or retail	2	3
Other (incl. design)	4	1
**Current Role Relevant to Project**		
Education programs manager/CEO	5	4
Dietitian/Nutritionist	3	0
Teacher/Principal	6	0
Canteen-related (manager/volunteer/association/committee)	4	4
End user (i.e., parent representative)	2	0
Other (e.g., marketing manager, founder)	1	3
**Parent/Caregiver**		
Yes	14	7
No	7	4
**State/Territory**		
SA	12	4
WA	3	3
NSW	2	1
VIC	3	2
TAS	1	1
QLD/NT/ACT	0	0

^1^ Includes three participants who contributed ideas, potential barriers and facilitators via written submission that did not participate in voting to rank ideas. ^2^ Multiple sectors could be selected per participant.

**Table 2:** Prioritized and ranked ^1^ food provision models from the consensus workshop.

Theme—Impact	Relative Importance (%) ^2^	Theme—Achievability	Relative Importance (%) ^2^
School lunch prepared onsite	43.9	School lunch prepared onsite	25.8
Community restaurant	18.2	School lunch prepared off-site (centralized)	19.7
Student/self-food preparation	15.2	Student/self-food preparation	18.2
Individual food boxes	9.1	Individual food boxes	16.7
School lunch prepared off-site (centralized)	9.1	Food trucks at schools	10.6
Food trucks at schools	4.5	Community restaurant	9.1

^1^ Ranking by impact and achievability. Only food provision models that received votes are presented. Three ideas did not receive votes for impact or achievability; these were healthy snack vending machines, classroom grazing station, and lunch at home. ^2^ Relative importance was calculated by ((score for each item)/(maximum points for the group, i.e., participant number × 6 points) × 100). Participants assigned 3 points to most preferred idea, 2 points to second most preferred, and 1 point to third most preferred.

**Table 3:** Top five ranked barriers to changing the school food system from the consensus workshop.

Barrier	Relative Importance (%) ^1^
Political barriers and lack of government support	27.3
Financial barriers and cost involved (e.g., staffing, products/service)	24.2
Change in infrastructure and equipment required (e.g., kitchen, dining)	19.7
Lack of resourcing (e.g., trained food preparation staff, teachers time for integrating with curriculum)	6.1
Implementation phase taking time and effort (e.g., people may not be willing or committed to making changes)	6.1
Level of support for change (including perceived value and need, fear of change)	6.1
Food preparation staff not being part of the school and valued	3.0

^1^ Relative importance calculated by ((score for each item)/(maximum points for the group, i.e., participant number × 6 points) × 100). Participants assigned 3 points to the most important barrier, 2 points to the second most important, and 1 point to the third most important.

**Table 4:** Top five ranked facilitators to changing the school food system from the consensus workshop.

Facilitator	Relative Importance (%) ^1^
Government support including cross agency and all political parties being committed	40.9
Tailored approach with variations for every type of school (e.g., small schools, regional schools with no kitchen)	13.6
Linking with external organizations, associations, sponsors or philanthropists working in the school food or nutrition space	13.6
Partnering with major retailers, primary production, suppliers and food relief agencies	7.6
School support including senior leadership and teachers	6.1
Consultation with all stakeholders during the process (e.g., students, teachers, parents, provides)	4.5
Having a pathway and retraining opportunities if canteen staff are no longer required	4.5

^1^ Relative importance calculated by ((score for each item)/(maximum points for the group i.e., participant number × 6 points) × 100). Participants assigned 3 points to the most important facilitator, 2 points to the second most important, and 1 point to the third most important.

## Supplementary Materials

The following are available online at https://www.mdpi.com/1660-4601/17/21/7935/s1, Table S1: Food provision model options categorized from idea generation workshops and discussions from the consensus workshop, Table S2: Potential barriers to changing the school food system ranked by importance at the consensus workshop (*n* = 11), Table S3: Potential facilitators to changing the school food system ranked by importance at the consensus workshop (*n* = 11).
