# Supplementary material for: Stakeholder Generated Ideas for Alternative School Food Provision Models in Australia Using the Nominal Group Technique
Source: Int J Environ Res Public Health. 2020 Oct 29;17(21):7935. doi: 10.3390/ijerph17217935 (PMC7663195; doi:10.3390/ijerph17217935)
Supplement: Supplementary file 1 [file ijerph-17-07935-s001.pdf]

Table S1. Food provision model options categorized from idea generation workshops and discussion from the consensus workshop .

Themed idea, description, and names of original ideas from idea generation workshops	Key discussion points from participants in the consensus workshop
**School lunch prepared onsite:**<br>**Idea A: School meal 'cafeteria' style (onsite food preparation)**<br>Food prepared on site by a cook/team of kitchen staff for a sit-down meal.<br>Parents pay a fee for the provision of a meal for their child, could be within school fees. Fee can be subsidized dependent on income and means testing.<br>Rotational menu (i.e., 20 days), 2 to 3 options and salad/veg bar, reflecting seasonal produce, minimally processed foods, and dishes representing different cultures.<br>OR/ parent-ordered choices<br>Teachers consuming the same food with students.<br>Eaten in a typical meal sharing environment (e.g., plates and cutlery, on a table), in a centralized area.<br>Students could be involved in washing up.<br>Could use canteen facilities.<br>**Synthesized from the following original ideas:**<br>Government-funded (with subsidies) cafeteria style<br>Food cooked onsite with food group approach and allocated time to eat lunch (at least 30 min) + seasonal foods<br>Food cooked onsite where possible and food consumed from plates with cutlery (reduce food packaging; environmental focus) + teachers eat with students<br>Food service model—foods ordered through school (produced on site) and delivered to/served in classroom<br>School café (cooked onsite, sit down meal)<br>School cooks (prepare school meals onsite, student participation in menu creation)<br>School meals prepared onsite (seasonal, minimally processed ingredients)<br>Community sit-down meal (meals reflecting cultures of school community)	This model could incorporate other ideas, such as Idea D where children could be involved in food preparation and school gardens<br>Important to consider the involvement of teachers, whether they eat with students or consume the same meal in a different location to enable conversations in the classroom after lunch.
**Individual food boxes:**<br>**Idea B: Individual food boxes (pre-prepared lunchbox, parent ordered)**<br>Food boxes delivered to the classroom/school/home for the week.<br>Parents can pre-order to accommodate any dietary requirements or cultural needs.<br>All boxes could be similar so students essentially getting the 'same' type of meal.<br>Families could access extended service for breakfast and dinner.<br>Could be government or for-profit run.<br>**Synthesized from the following original ideas:**<br>Parent-ordered, individual food boxes<br>Food delivery to school/home for the week (i.e., Lite & Easy model - could be Government or privately run; could extend to home meal delivery service)	This model could be used in addition to Idea A – e.g., if going on excursions etc.<br>Important to consider home vs school delivery of food boxes, in terms of student access and environmental impact.<br>Attempts to set-up this model in certain states were unsuccessful and competed with canteens and parents packing lunchboxes. However, this model may be more successful as a systematic approach rather than in addition to the current food provision system.
**School lunch prepared off-site (centralized):**<br>**Idea C: Centralized school meal delivered to schools (offsite food preparation)**<br>Commercial kitchens (e.g., secondary schools, TAFE campus) prepare and provide food to schools within their regions or jurisdiction.<br>Could contributes to those who are working towards accreditation in catering and/or hospitality.<br>Bulk prepared food delivered to schools to minimize costs and packaging.	Important to consider issues with food quality including nutrition, taste, presentation, and color; packaging/environmental impact; and options for regional schools where there may not be offsite kitchens/ facilities.

Themed idea, description, and names of original ideas from idea generation workshops	Key discussion points from participants in the consensus workshop
Could incorporate local growers, catering, business development.<br>**Synthesized from the following original ideas:**<br>Food cooked offsite (at a Hub) & delivered to schools<br>School Foodz (meal delivery to schools)<br>Food from community into school<br>Community kitchen in each school	Opportunities to learn from other setting such as hospitals using a centralized food preparation model, and to provide training/skills for disadvantaged young people to prepare meals.
**Optional add-on to School lunch prepared onsite:**<br>**Idea D: Student led/involved group food preparation (onsite) ***<br>Integrated with curriculum, +/- food production (e.g., fruit and vegetable garden, chickens, bakery)<br>Selected number of classes (e.g., 1-2) per day cooking simple meals to share with the rest of the school.<br>Within secondary schools could be run by students linked with business learning.<br>Eaten in a typical meal sharing environment (e.g., plates and cutlery, on a table).<br>Could include preparing ingredient take home boxes for families to pre-post purchase.<br>Could include a dish made in the classroom where students would bring designated ingredients.<br>Meals could be free or means tested.<br>**Synthesized from the following original ideas:**<br>Food grown in (school or community) garden, children grow, collect and cook<br>Food cooked onsite—children involved in the cooking process<br>Master mini chefs<br>Kids cafeteria (senior kids run cafeteria)<br>Students/classrooms involved in preparing foods at lunchtime<br>School food linking with the curriculum (children bringing food to prepare food together at school, deconstructing food and understanding why food is important)<br>Connecting food production/growing with eating (I.e. could be school garden, bakery, pickling, making products from scratch)<br>Japanese model of school food – children involved in whole ritual from serving to cleaning up linked with a kitchen garden<br>Pick your own lunch [>]	This model is not a standalone idea as still need a kitchen etc., and not sustainable therefore would be an add-on to Idea A.<br><br>Important to have students involved in food preparation to experience whole food system.<br><br>***This idea was incorporated with Idea A for voting***
**Student/self food preparation**<br>**Idea E: Student/self food preparation**<br>Students select and prepare their own lunch (e.g., salad bowl/sandwich) and snacks, before school (or in the classroom) at a food creation station or 'supermarket'. Foods could be divided into the food groups from the Australian Guide to Healthy Eating, with children selecting a certain number of items from each.<br>Items/ingredients may be semi-prepared but minimally processed (e.g., carrot sticks, cubed cheese).<br>Could be supported by food security sector. Could include food for before and after school.<br>Parents could pay a certain about per week/term/year for their children to access this food.<br>**Synthesized from the following original ideas:**<br>Food creation station (kids make own salads/sandwiches before school)<br>Food pack for the whole day (including food for before and after school)<br>Make your own [>]<br>Recess and lunch 'buffet' [>]	An example from community food nights for disadvantaged families, where parents and students collect foods (i.e. donations) and get lunch box ideas. Involving packing lunches at community food nights could be another option for how lunches are prepared.

Themed idea, description, and names of original ideas from idea generation workshops	Key discussion points from participants in the consensus workshop
**Community restaurant:**<br>**Idea F: Community restaurant (service multiple schools and community groups)**<br>Purpose built restaurant in the community (or on school grounds) to service multiple schools, senior centers, community groups.<br>Dedicated food preparation staff. Sit down meals and interaction between students and other community group members.<br>Planning menus could be part of class activities.<br>**Synthesized from the following original ideas:**<br>Purpose built restaurant outside of school servicing multiple schools, other community groups, seniors	This model provides a holistic approach to education and skill development across a range of areas, such as linking with healthy ageing, road safety (getting to restaurant), place for work (including for students on suspension). Likely a more expensive model but funding could be obtained from range of options given the holistic approach.<br>There are international examples where members of community came into the school and ate with students. Schools also prepared meals for nursing homes and the community.
**Idea G: Healthy snack vending machines**<br>Vending machines filled with healthy snacks, such as fresh cut, ready to eat produce, for access outside of the lunchbreak. Students could use pre-loaded passes to purchase items.<br>**Synthesized from the following original ideas:**<br>Vending machine (student pass with credits) for healthy snacks	This model is unlikely a stand-alone food provision option given the number of vending machines needed and daily restocking. Could be an add-on model when canteens are not available or open.<br>Important to consider packaging, freshness, quality, and costs to run refrigerated vending machines.<br>Opportunities to learn from hospitals that have great examples of fresh food vending machines.
**Idea H: Classroom grazing station**<br>Classroom grazing station in order of meals/time of day for children to access food as needed. Opportunity to integrate food with everyday education.<br>**Synthesized from the following original ideas:**<br>Classroom buffet / food integrated with classroom environment and part of everyday education<br>Platers to share ˃	This model could be a good option for snacks e.g. fruits and vegetables.<br>Important to consider the allergies and the risk cross contamination, nutritional quality of foods offered, impact of teaching grazing behaviors.
**Idea I: Food trucks at schools**<br>Food vendors come to schools each day, students can choose from menu of 3 to 4 options for a set price (rotating with schools in area every week or few days). Could be pre-ordered online the day prior.<br>**Synthesized from the following original ideas:**<br>Food Trucks (Different vendors, prepared fresh onsite)	This model could work well for smaller schools without access to kitchen, provides student learning opportunities (e.g., in menu develop and retail).<br>Important to consider if food trucks are government run and not privately owned as many existing food trucks have specialized menus and may need purpose designed menus.<br>Opportunities to learn from an example in one state where a canteen association has a purpose designed food truck that is used when training new canteen manages and has been used to cook with parents.
**Idea J: Lunch at home**<br>Students go home for lunch, can eat leftovers etc.<br>**Synthesized from the following original ideas:**<br>Eat at home ˃	Important to consider duty of care/supervision issue if parents are not at home during the day, and food access at home
**Idea K: Celebrity recipe branding**<br>Using celebrity branding on recipes to get students to try new recipes.<br>**Synthesized from the following original ideas:**<br>Celebrity recipe branding	Research team proposed that this is a strategy. Agreement from participants that this is strategy rather than a food provision model<br>Important to consider the risks as this strategy is used in junk food marketing which may make it difficult for children distinguish between junk food/healthy food and when to trust celebrity/ characters endorsing food items.<br>*** Idea removed from voting options***

* Curriculum links were mention in several ideas including school meals prepared on and off-site, whereas in this theme food preparation as part of the curriculum was a key element. ˃ Written submission

Table S2. Potential barriers to changing the school food system ranked by importance at the consensus workshop (*N* = 11).

Barrier	Total score [1]
Political barriers and lack of government support and understanding of the link between nutritious food and learning, social and health and wellbeing outcomes	18
Financial barriers and cost involved (including paying staff, overall cost of the products/service)	16
Change in infrastructure and equipment required (e.g., kitchen or dining facilities, physical space for a garden)	13
Lack of resourcing—staffing/personnel including trained food preparation staff, teachers having inadequate time for integrating with curriculum	4
Implementation phase—would take a lot of time and effort, people may not be willing or committed to making changes	4
Level of support for change including perceived value and need, and people not liking/fear of change	4
Food preparation staff not being part of the school and valued	2
Logistics—supply chains (including delivery, access to food, geographical location), seasonal availability, food storage and safety	1
Amplified barriers for regional schools (including lack of personnel and equipment, access to fresh ingredients including costs, shelf-life)	1
Relying on big business (e.g., profit focused), may not be healthy and tasty food, not building capacity in children or communities, food processers may have a 'vested' interest in having their food in schools	1
Cost cutting—processed foods will be seen as cheaper; perceived versus actual cost of processed foods	1
Decline overall of parent volunteers—lots of canteens have had to close /decrease because of parents not able to assist	1
Variation of funding in each state—state and/or Federal requirement	0
Time for preparation and capacity to feed all children in school	0
Children and schools are prime real estate (everyone wants to work in schools)	0
Food wastage and environmental friendliness	0
Existing contracts and procurement in place in schools	0
Canteens that are profitable (e.g., may be less support from parent body)	0
Differences in opinion as to what is 'healthy'	0
Poor problem identification—need to identify the right problem to solve it (e.g., what do parents identify as barriers?)	0
COVID-19 pandemic—the food industry may never be the same again	0
Cultural inclusivity and special diets—need to make sure food is culturally appropriate and meeting allergy needs, food intolerances	0
Lack of parental unity, interest and support (e.g., parents/teachers feel judged or threatened, not aware of long-term benefits)	0
Serious commitment from suppliers and manufacturers	0
Food fussiness/students' preferences	0
Future barrier—suggested ideas don't support the current 'canteen' model. Significant change management and cultural shift would be required to manage	0

[1] Participants voted 3-points to most important barrier, 2-points to second most important, and 1-point to third most important.

Table S3. Potential facilitators to changing the school food system ranked by importance at the consensus workshop ($N = 11$).

Facilitator	Total score [1]
Government support and backing of concept including support of cross agency work across all of government areas, and all political parties being committed to the long-term health of all Australians; and need a clear leader or position within government to lead school food agenda	27
Tailored approach with flexible models or variations in the model to have options for every type of school (e.g., small schools/large schools, regional schools with no kitchen or food prep space)	9
Linking with or getting buy-in from external organizations/sponsors or philanthropists working in the space—including government (state/federal and local about the need for a school food plan), consensus from the Federation of Canteens in Schools and State/Territory Canteen Associations and Networks; organizations supporting healthy eating in schools across Australia (i.e., kitchen garden programs, implementation programs)	9
Partnering with major retailers, primary production, suppliers for food supply and with other partner food providers or organizations (i.e., food relief)	5
School support of the concept, especially senior leadership, teachers/union (including providing education to promote interest)	4
Having a pathway if canteen managers are no longer required e.g., having employment in a new model, including retraining and having canteen staff part of the valued school staff	3
Consultation with all stakeholders during change process from ideas to implementation students, teachers, parents, providers etc.	3
Learning from what other countries have done and what worked and what didn't, including looking at the research	2
Dissemination and communication strategy—Excellent communication/media/public relations strategy to explain both the vision, practicalities and address objections, including highlighting the links between food and better child, adult, health care outcomes, and using passionate, focused results driven people who can connect with politicians	2
Connect and embed to the National Curriculum (e.g., could teach children cooking as well as nutrition) and use this to a marketing advantage	1
Ongoing long-term funding adequate for resourcing and food costs; ideally providing free food to increase uptake	1
Support from families/parents and students, including communicating with parents the benefits in terms of time and removing pressure	0
Student involvement and choice, including students voice in ideas for improvement, e.g., Children's Commissioner for Children and Young people	0
Teachers can act as role models	0
Partnering with equipment suppliers	0
Involvement from health professionals including dietitians, e.g., menu development, involved with training staff	0
Sourcing food grown at local level (i.e., feed children before the food is exported) which has growing interest since COVID-19 pandemic	0
Community organizations already focused on food production and service	0
Partnering with training organizations (e.g., TAFE)	0
Technology—apps, online ordering etc.	0
Off-site production including linking with local commercial kitchens	0
Taking a whole of school approach model as it has been shown to drive sustainable change	0
Taking a community and sustainability focus to bring in more players and solutions for the environment	0
Well managed change management process for Australia and school community	0
Public private partnerships (well managed)	0
Parental involvement / volunteering, e.g., family agreement when starting school for parents to participate and engage the school community	0
Convenience	0
Collective buying power of schools or group of schools	0

[1] Participants voted 3-points to most important facilitator, 2-points to second most important, and 1-point to third most important.
